# Systematic Review on the Mid-Term Outcomes of Elective Endovascular Aneurysm Sealing in Comparison to Endovascular Aneurysm Repair

**DOI:** 10.1177/15266028211047941

**Published:** 2021-09-27

**Authors:** Aleksandra C Zoethout, Iris Hochstenbach, Maarten J van der Laan, Jean-Paul P.M de Vries, Michel M.P.J. Reijnen, Clark J Zeebregts

**Affiliations:** 1 Department of Surgery, Division of Vascular Surgery, Universitair Medisch Centrum Groningen, Groningen, The Netherlands; 2 Department of Vascular Surgery, Rijnstate, Arnhem, The Netherlands; 3 Multi-Modality Medical Imaging Group, TechMed Centre, University of Twente, Enschede, The Netherlands

**Keywords:** aneurysm*, endovascular aneurysm repair, endovascular treatment/therapy, mortality, endoleak, migration, reintervention, systematic review

## Abstract

**Introduction:**

The Nellix endovascular aneurysm sealing (EVAS) system has been a topic of discussion. Early results were promising but did not deliver on the long-term and the device has been recalled from the market. This study compares literature for EVAS and conventional endovascular aneurysm repair (EVAR).

**Methods:**

A systematic review and analysis was conducted according to the preferred reporting items for systematic reviews and meta-analyses (PRISMA) guidelines. PubMed, Embase, and Cochrane Library were searched and identified the eligible studies. Proportion rates for the outcomes of interest were extracted. Subgroup analyses were performed for EVAS and EVAR.

**Results:**

A total of 12 studies were included (EVAS n = 4, EVAR n = 8) including 10,255 patients (EVAS n = 784, EVAR n = 9441). The longest duration of follow-up was 3.4 years for EVAS and 5.0 years for EVAR studies. Throughout follow-up the overall all-cause mortality rates were 6% for EVAS and 13% for EVAR, and endoleak of any type was described in 10% of EVAS and 17% of EVAR patients. The migration rate >10 mm was 8% for EVAS and 0% for EVAR and aneurysm growth >5 mm was found in 11% of EVAS and 3% of EVAR cases. Total reintervention rate was 13% for EVAS and 7% for EVAR patients. For all analyzed outcome parameters heterogeneity was >50%.

**Conclusion:**

There is a tendency toward lower mortality and overall endoleak rates for EVAS compared to EVAR but with a higher rate of migration, aneurysm growth, and reintervention. Despite lower overall endoleak rates there was a tendency toward less type II and more type I endoleaks after EVAS compared to EVAR. Substantial heterogeneity however limits robust statistical analyses, and is probably caused by significant instructions for use breach in EVAS-treated patients. We call for more high-quality and long-term follow-up studies on both EVAS and EVAR in order to confirm the trends found in this study.

## Introduction

Endovascular aneurysm sealing (EVAS) was developed in an attempt to reduce the complications that are seen after endovascular aneurysm repair (EVAR) including endoleak, migration, aneurysm growth, and secondary rupture and to decrease the subsequent need for reinterventions. Since its launch in 2013, the Nellix EVAS device has been a topic of discussion. A higher than anticipated mid-term migration rate led to a refinement of the instructions for use (IFU) in 2016. Subsequently, the device has been voluntarily recalled in 2019 and the Conformitè Europëenne mark suspended. A confirmatory investigational device exemption (IDE) trial, using the latest generation device and protocol and the refined IFU is currently being conducted (NCT03298477).

Early results of the initial IDE trial using Nellix were promising, with low incidences of migration, endoleak, and aneurysm growth through the first-year follow-up.^1,2^ After commercial release, further studies showed similar positive early results,^3^ especially for patients treated within the IFU and when procedural adequacy was met.^4^

Unfortunately, when studies started to look beyond the first 2 years after implantation, a higher than anticipated complication rate was observed. Device related complications were seen in up to 26% of cases^5^ including distal migration of the endograft, type Ia endoleak, and secondary sac rupture.^6,7^ Nevertheless, trials with EVAS showed a lower overall all-cause mortality compared to traditional EVAR, potentially related to less cardiac-related deaths.^6^ However, more studies with long-term follow-up reported high rates of device failure and reintervention beyond 2 years.^7^

Before robust conclusions can be drawn, a comparison between EVAS and EVAR must be made to ascertain their differences in outcome. The aim of this study was to systematically review and compare literature regarding outcomes after EVAS and EVAR used for the elective treatment of abdominal aortic aneurysms (AAAs) with at least 2 years of follow-up. A comparison will be made between results including device related complications, reintervention rate, and all-cause mortality.

## Methods

This review was executed and reported using the preferred reporting items for systematic reviews and meta-analyses (PRISMA) guidelines.^8^ Prior to commencement of literature search, the study was registered at the prospective register of systematic reviews (PROSPERO 187639). The investigated endpoints in this study were; mortality, endoleak, migration, aneurysm growth, reintervention, aneurysm rupture, and stent-graft occlusion.

### Search Strategy

Pubmed, Embase, and Cochrane libraries were systematically searched from the first through the May 28th, 2020. Relevant studies were selected using the following word search in title and abstract: “abdominal aortic aneurysm” AND (“endovascular aneurysm sealing” OR “endovascular aneurysm repair”) AND (“treatment outcome” OR “mortality” OR “postoperative complications” OR “endoleak” OR “ruptured aneurysm” OR “migration” OR “stenosis” OR “occlusion” OR “aneurysm growth”). Studies were only selected if they were cohort, prospective, observational, and/or longitudinal studies published after 2010. An extensive description of search strings for all literature databases can be found in Appendix 1.

### Selection Criteria and Study Selection

After the literature search was completed and deduplication was performed, 2 independent researchers (A. Zoethout and I. Hochstenbach) manually reviewed the studies titles and abstracts on eligibility criteria. Studies were included for full-text evaluation only if they complied with the following criteria. First, they needed to present results from patients with unruptured, asymptomatic, and infrarenal AAA undergoing elective repair with EVAS or EVAR without adjunctive procedures (eg, fenestrated endografts, chimney or snorkel procedures, or the use of endoanchors). All EVAS and EVAR devices were selected to have an implantation date from January 2010 onward in order to provide relevant and currently used devices and techniques and because EVAS was introduced for investigational use at this time. Studies needed to report on at least one of the outcome measures of interest; reintervention, mortality (aneurysm and all-cause mortality), and device related complications (including migration, endoleak, aneurysm growth, aneurysm rupture, stenosis, and occlusion). Reviews and animal studies were excluded from the study, as were studies with a mean or median follow-up duration of less than 2 years. Finally, studies were excluded if they were not written in English. In case of discrepancy between included articles, the reviewers reached consensus by discussion.

Included manuscripts were then retrieved and screened on full-text in a similar manner to make a final selection for inclusion. In cases where studies were performed based on the same database, the retained study would be the latest publication, publication with larger sample size, or publication with the longest follow-up.

### Data Collection and Analysis

Data were extracted from included articles by 2 reviewers (AZ and IH) using a predesigned data collection form. Methodological quality of included studies was assessed by 2 independent reviewers (AZ and IH) using the Newcastle-Ottawa Scale (NOS) for observational studies.^9^

The Freeman-Tukey double arcsine transformation was used to estimate effect sizes and their 95% confidence intervals (CI) from the presented proportion rates. A random-effects model was used to pool the results of each outcome parameter and forest plots were created comparing EVAR and EVAS. In order to estimate heterogeneity, *I*^2^ was calculated. If *I*^2^ > 50%, heterogeneity was deemed too great and no comparative pooling was performed. Instead, results will be presented as group averages and a forest plot will graphically support the results. All statistical analyses were performed using Stata 16 software (StataCorp LP).

## Results

### Study Selection

**Figure 1.**
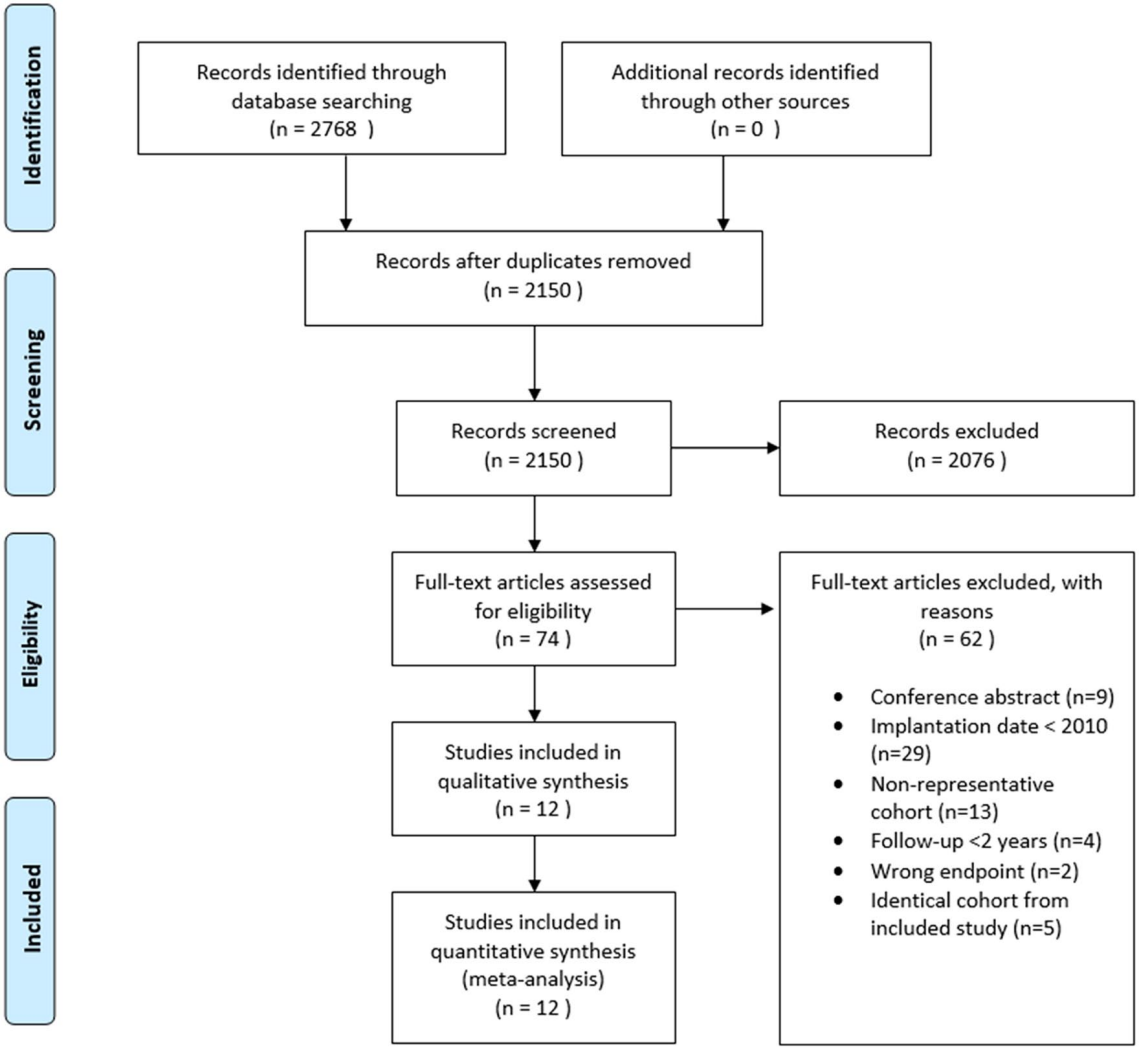
PRISMA flow diagram of study selection.

The literature selection process is summarized in a PRISMA flow diagram in Figure 1. A total of 2768 studies were identified through literature search, 2150 remained after duplicate removal. After screening on title and abstract, 74 articles were screened on full-text. Of these, 62 were excluded for the following reasons; conference abstracts (n = 9), implantation date before 2010 (n = 29), follow-up <2 years (n = 4), reported on an identical cohort as an included article (n = 5), reported on other outcomes (n = 2) or had a non-representative cohort (n = 13) most commonly by including symptomatic AAA’s in their cohort. After completion of screening, 12 articles were included in the systematic review including 8 studies reporting on EVAR and 4 on EVAS. The quality of the studies is displayed in Table 1 by means of the NOS.^9^ Nine out of 12 studies were non-comparative studies and all studies had adequate length of follow-up, but most studies failed to report on loss of follow-up.

**Table 1.** Newcastle-Ottawa Scale with Number of Stars Awarded Per Study Per Domain.^5,10–20^

	Representativeness of the exposed cohort	Selection of thenon-exposed cohort	Ascertainment of exposure	Outcome of interest was not present study start	Comparability of cohorts based on design or analysis	Assessment of outcome	Long enough follow-up for outcomes to occur	Adequacy of follow-up of cohorts
EVAR								
Bonfill, 2019^19^	*	*	*	*	**	*	*	
Deery, 2018^17^	*		*	*	*	*	*	*
Howard, 2018^10^	*		*	*	*	*	*	
Massière, 2020^11^	*	*	*	*	*	*	*	
Pinto Sousa, 2017^18^	*		*	*	*	*	*	
Sirignano, 2018^12^	*		*	*	**	*	*	
Symonides, 2018^20^	*	*	*	*	*	*	*	
Torsello, 2019^13^			*	*	*	*	*	*
EVAS								
Carpenter, 2018^16^			*	*	*	*	*	
Harrison, 2018^5^	*		*	*	*	*	*	
van Noort, 2018^14^	*		*	*	*	*	*	
Yafawi, 2019^15^			*	*	*	*	*	

Abbreviations: EVAR, endovascular aneurysm repair; EVAS, endovascular aneurysm sealing

* = point awarded

### Study Characteristics

A total of 10,225 patients were included of whom 9441 underwent EVAR and 784 EVAS.^5,10–20^ The complete study population consisted largely of men (88.9%) with a mean age of 74.8 ± 7.9 years. A higher age was observed in the EVAS patients (p < 0.0001) with a mean age for the EVAS cohort of 75.9 ± 7.9 and 74.2 ± 7.9 years for the EVAR cohort. The longest duration of follow-up was 5.0 years for EVAR and 3.4 years for EVAS studies. A summary of study characteristics and reported outcomes is presented in Table 2.

**Table 2.** Overview of Study Characteristics and Outcome Measures Reported per Included Study.^5,10–20^

	Country	Number of patients	% male	Mean age, y(range)	Mean follow-up, y	Mortality	Endoleak	Migration	Aneurysm growth	Aneurysm rupture	Stenosis and occlusion	Reintervention
EVAR												
Bonfill, 2019^19^	Spain	87	94.3	76	2.9	X						
Deery, 2018^17^	USA	178	82.0	NR	3.1	X	X	X		*	*	X
Howard, 2018^10^	Australia, UK, and Germany	3166	85.6	73	5	*	*	*		*		*
Massière, 2020^11^	Brasil	203	90.1	73	2.9	*	*				*	X
Pinto Sousa, 2017^18^	Portugal	56	96.4	78	3.4	X	X	X		*		
Sirignano, 2018^12^	Italy	306	90.5	73	2.9	X	*		X		*	X
Symonides, 2018^20^	Poland	5469	86.1	72	2.3	X						
Torsello, 2019^13^	Global	60	95.0	74	5	X	*	X	X	*	*	*
EVAS												
Carpenter, 2018^16^	Global	333	93.7	73	2.1	X	X	X	X	*	*	X
Harrison, 2018^5^	UK	115	NR	79	3.4	*	*	*		*		
van Noort, 2018^14^	The Netherlands	261	87.7	76	2		X	*	X			
Yafawi, 2019^15^	UK	75	76.0	76	2	X	X	X	X	*		X

Abbreviations: NR, not reported; UK, United Kingdom; USA, United States of America; X, outcome reported included in analysis.

* outcome reported but not suitable for inclusion in analysis.

### Mortality

**Figure 2.**
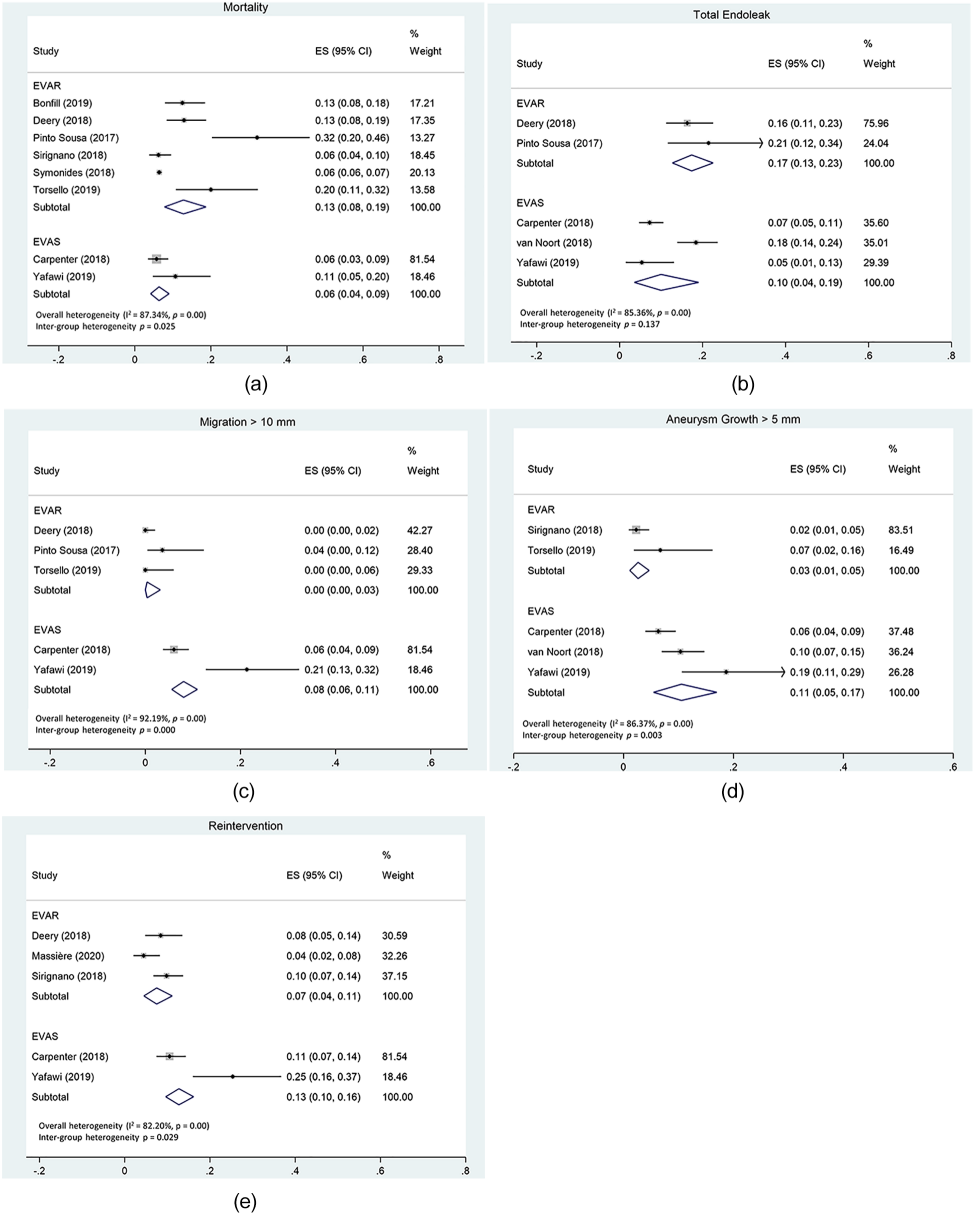
Forest plots for different outcome parameters with subgroup analysis for EVAS and EVAR. (2A) Forest plot for mortality. (2B) Forest plot for total endoleak. (2C) Forest plot for migration >10 mm. (2D) Forest plot for aneurysm growth. (2E) Forest plot for reintervention.

In total, 8 studies reported analyzable data on all-cause mortality, of which 2 studies looked at EVAS and 6 at EVAR. There was significant and considerable overall heterogeneity (p < 0.00 with *I*^2^ = 87%) and significant inter-group heterogeneity (p = 0.025) between EVAS and EVAR. Therefore overall pooling of results was not performed. All forest plots can be found in Figure 2, including all-cause mortality in Figure 2A.

The total all-cause mortality rate in EVAS studies was 6% (95% CI: 4%–9%). Additionally, Harrison et al^5^ reported 52 deaths during follow-up. This however concerned the combined cohort of 161 patients, including non-elective and non-conventional EVAS procedures and therefore was not included in the analysis. For EVAR, overall all-cause mortality was 13% (95% CI: 8%–19%) at total follow-up.

### Endoleak

Most studies who reported endoleak, were only reported on overall endoleak and did not stratify into endoleak types. The 3 EVAS and 2 EVAR studies were included in the forest plot (Figure 2B). There was considerable overall heterogeneity present (p = 0.00 with *I*^2^ = 85%). Inter-group heterogeneity was not observed (p = 0.137).

For EVAS, the total endoleak rate was 10% (95% CI: 4%–19%). Harrison et al^8^ did not report on all types of endoleak, but noted that of the 8 aneurysm ruptures after EVAS, 7 were secondary to a type Ia endoleak and the other rupture was associated with a type Ib endoleak. In addition, of the 5 device failures which occurred in the first year after EVAS, 3 were caused by type Ia endoleak and 2 by type Ib endoleak.

The total endoleak rate for EVAR was 17% (95% CI: 13%–23%). Howard et al^10^ only provided 30 day follow-up endoleak rates, totaling to 25 cases with endoleak (0.01%). Throughout long-term follow-up, the freedom from type Ia endoleak was reported in 99% and 97% at 1 and 5 years follow-up, respectively. Massière et al^11^ reported on long-term prevalence of type I endoleak and noted 2 cases (1%) of type Ia endoleak and 5 cases (2%) of type Ib endoleak. Sirignano et al^12^ reported 6 cases of type Ia endoleak (2%), 3 type Ib endoleak (1%), 7 type II endoleak (2%), and 1 type III endoleak (0.3%). Torsello et al^13^ reported the prevalence of endoleak as cumulative numbers and could therefore not be included in the forest plot since patients were probably counted double. They noted 2 type Ia and 2 type Ib endoleaks (3.3%), 31 total cases of endoleak type II (53%) at 1 month follow-up and 10 cases (26%) at 5 years follow-up.

### Migration

The total of 2 EVAS studies and 3 EVAR studies were included in the analysis on graft migration (Figure 2C). All but 1 study^14^ used >10 mm migration as their definition^13,15,16^ or did not specify their cut-off value for migration.^17,18^ For the analysis, only the migration of >10 mm was used in order to facilitate comparison, for those studies which did not specifically define migration it was assumed they used the most standard value of >10 mm. Overall heterogeneity was *I*^2^ = 92% (p < 0.00) and inter-group heterogeneity was also significant (p < 0.00).

For EVAS, the total migration rate was 8% (95% CI: 6%–11%). Additionally, the other 2 EVAS studies, by Harrison et al^5^ and Van Noort et al,^14^ also reported on graft migration. Harrison et al^5^ noted device failure in 42 of 115 patients (37%), mainly caused by caudal migration of the stents. Van Noort et al^14^ found a total of 57 cases of migration (22%), of which 12 cases were graft migration in combination with any type of endoleak and 13 cases a combination of migration, endoleak, and aneurysm growth. The definition of graft migration used in the study of Van Noort et al^14^ was ≥5 mm, and was therefore not included in the forest plot.

The total migration rate for EVAR was 0% (95% CI: 0%–3%). However, Howard et al^10^ mentioned 1 case of graft migration in their cohort of 3166 patients (<0.00%) within 30 days follow-up, but did not mention graft migration throughout long-term follow-up.

### Aneurysm Growth

Aneurysm growth was reported in 3 EVAS and 2 EVAR studies (Figure 2D). The definition was growth of >5 mm compared to maximum preoperative AAA diameter. Only 1 study used aneurysm growth of ≥5 mm as their definition but was nevertheless included in the analysis,^15^ this study also had the highest incidence of aneurysm growth. There was considerable overall heterogeneity with *I*^2^ = 86% (p < 0.00) and significant inter-group heterogeneity (p < 0.00). For EVAS, the total percentage of aneurysm growth was 11% (95% CI: 5%–17%). The total aneurysm growth for EVAR was 3% (95% CI: 1%–5%). All cases of aneurysm growth were related to endoleak type II and reintervention was needed for every reported case.

### Reintervention

The total of 2 EVAS and 3 EVAR studies were included in the forest plot of reintervention (Figure 2E). There was substantial overall heterogeneity with *I*^2^ = 82% (p < 0.00) and significant inter-group heterogeneity between EVAR and EVAS studies (p = 0.03).

The total reintervention rate for EVAS was 13% (95% CI: 10%–16%). The 2 other studies on EVAS by Harrison et al^5^ and Van Noort et al^14^ did not report reintervention rates.

For EVAR, total reintervention rate was 7% (95% CI: 4%–11%). The 2 EVAR studies reporting on reintervention were not included in the forest plot. Howard et al^10^ reported 89 reinterventions (3%) within the first 30 days of follow-up and a 93.7% and 83.2% freedom from any aortic related reintervention at 1 and 5 years respectively. Torsello et al^13^ mentioned 2 reinterventions for 2 cases of type Ia endoleak, but did not provide a total number of reinterventions throughout follow-up.

### Other Outcome Parameters

Aneurysm rupture was reported in 3 EVAS studies. There were 2 early deaths due to rupture reported by Carpenter et al .^16^ and 1 additional rupture leading to secondary intervention during follow-up, leading to a total of 3 (1%) secondary ruptures. Harrison et al^5^ reported 8 patients (7%) with secondary aneurysm rupture, of whom 6 patients experienced rupture 2 years post EVAS and all but 1 rupture occurred in the presence of type Ib endoleak. Yafawi et al^15^ noted 2 (3%) asymptomatic aneurysm ruptures through the first 30 days of post-operative and gave no further information on secondary rupture at longer follow-up. The 4 EVAR studies mentioned aneurysm rupture but reported zero cases secondary rupture throughout follow-up.^10,13,17,18^

Occlusion was mentioned in 2 EVAS studies. Carpenter et al^16^ described 35 reinterventions, of which 17 (49%) were due to limb occlusion. Yafawi et al^15^ did not report on the outcome occlusion, however, they noted 5 cases of limb ischemia amongst the 30 days complications. For EVAR, 3 studies reported on occlusion. Deery et al^17^ reported graft limb occlusion in 2.3% of patients. Massière et al^11^ reported 2 (1%) cases of occlusion within the perioperative period. Sirignano et al^12^ noted that out of the 20 reinterventions needed at some point during the 35 months follow-up, 7 were due to endograft limb occlusion. Finally, Torsello et al^13^ noted 1 patient with occlusion after 3 years, without reintervention throughout 5 years.

## Discussion

When assessing outcome parameters, all-cause mortality had most available data. There is a tendency toward lower all-cause mortality rates after EVAS (6%) compared to EVAR (13%). Previous studies^6,21^ have shown that EVAS leads to a decreased inflammatory response, potentially related to the active sac management. O’Donell et al^6^ have reported a significantly better survival after EVAS, comparing 333 patients in the Nellix United States Investigational Device Exemption trial to 15,431 controls from the vascular quality initiative in a propensity weighted approach.^6^ In the current study the EVAR studies tended to have longer follow-up time which may have skewed the mortality rates negatively toward EVAR.

Several reasons exists for the tendency toward a lower incidence of endoleak after EVAS (10%) compared to EVAR (17%). Due to the sac-sealing design, EVAS is protected from type II endoleaks which was confirmed by the study by Yafawi et al.^15^ However, Carpenter et al^16^ did observe 11 cases of type II endoleak (3.3%), but all of these were of a very low volume. The type II endoleak incidence after EVAR appears to be higher than after EVAS, based on the studies found. Contrarily, type Ia endoleak appears to be a problem with EVAS, with 1 study even reporting a freedom from type IA endoleak as low as 61.4% at 5 years.^22^

Graft migration is a major problem with EVAS and is confirmed in our study with almost no cases of migration >10 mm amongst EVAR patients, but an 8% migration rate within the EVAS studies. The EVAS system was developed to obtain its fixation from the polymer-filled endobags without active fixation using proximal or distal hooks. After curing of the polymer, the endobags are filled and its dimensions are fixed, however, arteries and aneurysms appear to adapt and change over time.^22,23^ In order for EVAS to be reintroduced on the market, improvement in proximal seal appears to be crucial. The trend toward a higher rate of migration after EVAS compared to EVAR is likely related to the tendency of higher prevalence of type Ia endoleak for EVAS.

Aneurysm growth was related to type II endoleak in all EVAR cases, which is an established correlation.^24^ As mentioned before, type II endoleak hardly occur after EVAS. Contradictorily, there was a trend toward more aneurysm growth after EVAS (11%) compared to EVAR (3%). It must be noted, however, that the 1 EVAS study^15^ with the highest rate of growth of all studies used the definition of ≥5 mm growth whereas the other studies used >5 mm growth. EVAS migration and possible subsequent endoleak type Ia has been observed to be a high risk for aneurysm growth.^25^ It should be noted that in the first commercial generation of EVAS the endobag had no distal fixation, and consequently the endobag could shift upwards, compromising the distal seal. The trend toward a higher reintervention rate after EVAS (13%) compared to EVAR (7%) could be due to the above mentioned problems of migration, type Ia endoleak and aneurysm growth. Additionally, a learning curve with the new EVAS device might have played a role. The relatively unpredictability of the proximal edge of the endobag may have caused a low deployment of the endografts.

There were several limitations to this study, the major being heterogeneity. Despite strict inclusion criteria, substantial clinical and statistical heterogeneity was observed which made pooling of the results and comparison between the groups statistically invalid. A priori planned subgroup analysis of EVAS and EVAR studies could not explain the heterogeneity, since there was not enough data present to analyze. The main cause for the heterogeneity is that IFU adherence was different between the 2 groups. Of the 784 included EVAS patients in this study, 153 patients (20%) were treated outside the original 2013 IFU and 568 patients (72%) were treated outside the refined 2016 IFU. Nonadherence to IFU was markedly lower in the EVAR studies, at only 2%^17^ and 13%.^10^ It is likely that this was because the aortic anatomy of the EVAS population was markedly more challenging. This is confirmed by Harrison et al,^5^ who noted that of the patients undergoing EVAS, 25% of patients had no other endovascular repair option and only 46% of patients were suitable for conventional EVAR.

Another limitation of the study is that the longest duration of follow-up was substantially longer for EVAR and this might have negatively influenced the results after EVAR. Additionally, for the EVAR studies different devices were used and 4 of the studies did not mention the specific device implanted. As can be expected with a relatively new device, there are less studies published to date on EVAS than there are for EVAR. This is also reflected in our comparison of 4 included EVAS studies against 8 EVAR studies. Throughout our review, we found no randomized trials for EVAS devices matching our inclusion criteria. As such, we excluded randomized EVAR trials in order to increase homogeneity in our analysis. Thereby, we are aware that the strength of evidence is reduced. Additionally, it would have been preferable to include studies with more long-term follow-up however, this study was limited to the manuscripts available at the time of review. Also, publication bias might be in place for this study, which can result in overestimation of the treatment effect. For example, even though the Nellix device has been introduced to the market 7 years ago, only 4 studies with follow-up of 2 years or more were found. Publication bias could not be statistically demonstrated because less than 10 studies were present in each analyzed outcome parameter.^26^ Finally, the risk of bias found in included studies was moderate, with only 2 studies having an adequate report of loss of follow-up. It is plausible that the patients lost through follow-up did not have complications. This could affect the long-term results negatively and overestimating the complication rate.

## Conclusion

This systematic review and literature analysis showed that there is a tendency toward lower mortality and overall endoleak rates for EVAS compared to EVAR but with a higher rate of migration, aneurysm growth, and reintervention. Despite lower overall endoleak rates there was a tendency toward less type II and more type I endoleak after EVAS compared to EVAR. Substantial heterogeneity however limits robust statistical analyses, and is probably caused by significant IFU-breach in EVAS-treated patients. This study calls for more high-quality studies with long-term follow-up as well as comparing studies for EVAS and EVAR.

## Appendix I: Search Strategy

### PubMed

(“Aortic Aneurysm, Abdominal”[Mesh] OR “abdominal aortic aneurysm*”[tiab] OR aaa[tiab])

AND

(“endovascular aneurysm sealing”[tiab] OR evas[tiab] OR “endovascular aneurysm repair*”[tiab] OR evar[tiab])

AND

(“Treatment Outcome”[Mesh] OR “treatment outcome*”[tiab] OR Reoperation[mesh] OR reoperat*[tiab] OR Mortality[Mesh] OR mortal*[tiab] OR “Postoperative Complications”[Mesh] OR “postoperative complicat*”[tiab] OR endoleak*[tiab] OR “Aneurysm, Ruptured”[Mesh] OR “aneurysm ruptur*”[tiab] OR “ruptured aneurysm*”[tiab] OR migrat*[tiab] OR stenos*[tiab] OR occlusion[tiab] OR “aneurysm grow*”[tiab] OR “sac expansion”[tiab])

AND

(“Cohort Studies” [Mesh] OR cohort[tiab] OR prospective[tiab] OR observational[tiab] OR longitudinal[tiab])

Used filter: publication date 2010 onward

### Embase

(“Abdominal Aortic Aneurysm”/exp OR “abdominal aortic aneurysm*”:ab,ti OR aaa:ab,ti)

AND

(“Aneurysm sealing system”/exp OR “endovascular aneurysm sealing”:ab,ti OR evas:ab,ti OR “endovascular aneurysm repair”/exp OR “endovascular aneurysm repair*”:ab,ti OR evar:ab,ti)

AND

(“Treatment Outcome”/exp OR “treatment outcome*”:ab,ti OR “Reoperation”/exp OR reoperat*:ab,ti OR “Mortality”/exp OR mortal*:ab,ti OR “Prosthesis Complications”/exp OR “prosthesis complicat*”:ab,ti OR endoleak*:ab,ti OR “aneurysm rupture”/de OR “aneurysm ruptur*”:ab,ti OR “ruptured aneurysm*”:ab,ti OR migrat*:ab,ti OR stenos*:ab,ti OR occlusion:ab,ti OR “aneurysm grow*”:ab,ti OR “sac expansion”:ab,ti)

AND

(“Cohort Analysis”/exp OR cohort:ab,ti OR prospective:ab,ti OR observational:ab,ti OR longitudinal:ab,ti)

Used filter: publication date 2010 onward

### Cochrane Library

(“abdominal aortic aneurysm*” OR aaa)

AND

(“endovascular aneurysm sealing” OR evas OR “endovascular aneurysm repair*” OR evar)

AND

(“treatment outcome*” OR reoperat* OR mortal* OR “postoperative complicat*” OR endoleak* OR “aneurysm ruptur*” OR “ruptured aneurysm*” OR migrat* OR stenos* OR occlusion OR “aneurysm grow*” OR “sac expansion”)

AND

(cohort OR prospective OR observational OR longitudinal)

Used filter: publication date 2010 onward, trials only
